# Post-trauma morbidity, measured as sick leave, is substantial and influenced by factors unrelated to injury: a retrospective matched observational cohort study

**DOI:** 10.1186/s13049-017-0444-3

**Published:** 2017-10-13

**Authors:** Erik von Oelreich, Mikael Eriksson, Olof Brattström, Andrea Discacciati, Lovisa Strömmer, Anders Oldner, Emma Larsson

**Affiliations:** 1 Perioperative Medicine and Intensive Care, Karolinska University Hospital, Solna, SE-171 76 Stockholm, Sweden; 2 Section of Anaesthesiology and Intensive Care Medicine, Department of Physiology and Pharmacology, Karolinska Institute, Stockholm, Sweden; 3 Unit of Biostatistics, Institute of Environmental Medicine, Karolinska Institute, Stockholm, Sweden; 4 Division of Surgery, Department of Clinical Science, Intervention and Technology (CLINTEC), Karolinska Institute, Stockholm, Sweden

**Keywords:** Trauma, Sick leave, Outcome, Injury severity

## Abstract

**Background:**

Mortality as an endpoint has been the focus of trauma research whereas few studies investigate long-term outcomes in terms of morbidity. An adequate analysis of post-injury morbidity includes several dimensions, for this reason sick leave has been used as a proxy for morbidity in the current study. The aim of this retrospective matched observational cohort study was to investigate sick leave before and after trauma and factors associated with prolonged sick leave.

**Methods:**

Patients from a level one trauma centre 2005–2010 were matched in a 1:5 ratio with uninjured controls. By linkage to national registries, sick leave rates were compared. The association between potential risk factors and full-time sick leave at twelve months post injury, the primary end-point, was examined in trauma patients by logistic regression.

**Results:**

Four thousand seven hundred twelve patients and 25,013 controls aged 20–63 were included. Trauma patients had more sick leave both before and after trauma. Age, psychiatric disease, low level of education, serious injury, spinal injury, reduced consciousness at admission, discharge destination other than home, and hospital length of stay >7 days were all associated with the primary end-point. The strongest risk factor was sick leave before trauma; this was also noted in the most seriously injured patients.

**Discussion:**

In this retrospective matched observational cohort study we found a significant long-term morbidity, measured as sick leave, among trauma patients. Compared to controls the difference was maximal early after trauma and sustained throughout the follow up period. In the logistic regression, factors associated with the traumatic injury as well as host factors increased the probability of not returning to work. Full sick leavemonth twelve post injury was strongly associated with pre-injury sick leave but also with age, psychiatric comorbidity, level of education, injury severity, spinal injury, low GCS at admission, length of stay at hospital and discharge to other destination than home.

**Conclusions:**

Trauma patients suffer from significant long-term morbidity. The sustained post-trauma morbidity is largely influenced by factors not related to injury per se. These insights enable identification of patients at risk for prolonged sick leave after trauma.

## Background

The overall goal of initial trauma care is the survival of the injured patient, but in a long-term perspective the return to an independent life is of outmost importance for the individual as well as for society. The burden of sick leave on society is important, but often foreseen in the aims of improving trauma care. In the literature, mortality after trauma is relatively well studied whereas the number of studies investigating long-term outcomes in terms of morbidity is limited [1]. In addition to severity of injury, several factors seem to influence the degree of morbidity after trauma [2]. It is complicated to access post-injury morbidity over time for a large cohort. In this context, return to work has been used as a proxy for morbidity. The majority of previous studies investigating risk factors associated with delayed return to work are interview based and commonly restricted to small samples without any quantitative measurement of sick leave [3–8]. One of few larger previous studies showed a significant degree of disability among trauma victims two years after injury [2].

We hypothesized that, in addition to injury severity, educational level and comorbidity would significantly influence post-trauma sick leave. The aim of the current study was to investigate the magnitude of sick leave both before and after trauma, and to identify risk factors for the end point full sick leave the twelfth month after trauma.

## Methods

### Setting and study population

In this retrospective matched observational cohort study patients in the Trauma Registry at the Karolinska University Hospital, Stockholm, Sweden, with a first trauma admission between January 2005 and December 2010 were eligible for inclusion. All patients ≥ 15 years of age admitted with trauma team activation, regardless of Injury Severity Score (ISS), as well as patients admitted without trauma team activation but found to have an ISS > 9, are consecutively included in Trauma Registry Karolinska. Our institution is a level one trauma centre and the only referral centre for trauma patients from the greater Stockholm region of approximately two million inhabitants. Patients with isolated fractures of the upper or lower extremity, drowning, chronic subdural hematomas, burn injuries and hypothermia without concomitant trauma are not included in Trauma Registry Karolinska.

Below the age of 20 years a limited number of individuals have full time work and they were therefore excluded from the study. The retirement age is generally 65 years, therefore only individuals <64 years of age at the time of trauma were included since they otherwise were unlikely to return to work. Individuals receiving 100% disability pension (since 2003 replaced by *activity compensation* for individuals between 19 and 29 years and *sickness compensation* between 30 and 64 years [9]) at the time of trauma were excluded since they were unlikely to return to work. In the national health insurance system, all individuals including students, are entitled to payed sick leave in case of sickness regardless of employment status. Thus, unemployed individuals were also included in the study. Patients without a valid personal number (e.g. individuals without a Swedish citizenship or newly arrived immigrants, *n* = 359) were excluded since information regarding pre-trauma exposure is not available. In total 7382 trauma patients were included in the study.

Eligible controls were Swedish residents not found in Trauma Registry Karolinska. Every injured patient was matched to five controls who were of the same age and sex, and registered in the same municipality on January 1 in the year of index trauma. A random sample from the general population of 36,910 age-, gender- and municipality-matched controls were extracted from the Total Population Register (managed by Statistics Sweden). One hundred and fifty controls dying before the trauma date for their respective case were omitted yielding a total of 36,759 controls.

**Fig. 1:**
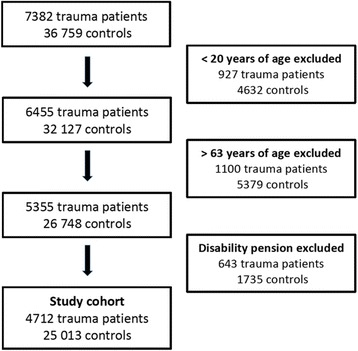
Flow chart of included patients

Patients and controls were excluded if they were younger than 20 (*n* = 927 and *n* = 4632 respectively) or older than 63 years of age (*n* = 1100 and *n* = 5379 respectively), yielding 5355 trauma patients and 26,748 controls. In addition, patients and controls were excluded if they received disability pension prior to trauma (*n* = 643 and *n* = 1735 respectively). In total, 4712 trauma patients and 25,013 controls were included in the study (Fig. 1). The full cohort was followed for at least one year with a median follow up time of three years and seven months. Time zero for both patients and controls was the date of index trauma.

The study adhered to the Strengthening the Reporting of Observational Studies in Epidemiology (STROBE) recommendation for cohort studies [10].

### National registries

All Swedish citizens have a unique personal identification number that enables linkage between different registries [11]. The Swedish National Patient Register managed by the National Board of Health and Welfare (NBHW) covers information regarding public inpatient and outpatient visits [12]. Each care episode is classified according to the International Classification of Diseases (ICD-10). The Cause of Death Register, managed by NBHW, records cause and time of death for all deceased Swedish residents. Statistics Sweden is responsible for official statistics regarding the national census and manages the Total Population Register.

### Exposure and outcome ascertainment

Data on comorbidity were retrieved from the National Patient Register. Comorbidity was assessed up to eight years prior to trauma. Somatic comorbidity was defined as the presence of any of the somatic diagnoses included in the Charlson Comorbidity Index modified to ICD-10 [13]. Psychiatric comorbidity was defined as the presence of a diagnosis in ICD-10 groups F20-F99 and substance abuse as a diagnosis in F10-F19 respectively. Data on education were extracted from the Total Population Register. Education was classified into low, medium and high, representing ≤9, 10–12 and >12 years (last category equaling university level) of schooling respectively. Trauma-related data, including injury mechanism and injury severity, were extracted from Trauma Registry Karolinska. Treatment-related information such as length of stay (LOS) and discharge destination was also extracted from the Trauma Registry. Injury severity was classified according to the Injury Severity Score based on the Abbreviated Injury Scale (AIS) 1990 edition for year 2005–2006 and AIS 2005 edition from 2007. Serious injury to an AIS-region was defined as AIS > 2 and serious injury in general as ISS > 15. Deceased individuals were identified in the Cause of Death Register.

The Swedish social insurance system provides compensation for sick leave and is administered by the Swedish Social Insurance Agency. Sick leave can be part time or full time. Thus, one net day could be composed by one day of 100% sick leave or four days of 25% sick leave. The first day of sick leave is unpaid, the employer pays the following thirteen days and after that the Swedish Social Insurance Agency is responsible for compensation. Hence, not all episodes less than two weeks are recorded in the Swedish Social Insurance Agency database. Thus, in the current study records for sick leave for the first month following trauma are incomplete and therefore not included in the analysis. Sick leave is presented as mean days/month per person. Full sick leave month twelve after trauma, the primary endpoint in the logistic regression analysis, was defined as full sick leave month twelve after trauma. Disability pension provided *after* the trauma was considered equal to sick leave in the analysis.

### Statistics

Categorical data are presented as proportions and percentages and continuous data with median and interquartile ranges except for days spent on sick leave which are presented as means. Crude comparisons of proportions were performed by using chi-square tests.

First, we estimated the crude differences in mean number of days spent on sick leave according to trauma/control status over time using a Generalized Estimating Equation (GEE) regression model with an identity link function and an exchangeable working correlation matrix [14, 15]. Given the longitudinal nature of the data, we employed GEE to account for the potential correlation among the number of days spent on sick leave within subjects over time. The GEE regression model included a trauma/control indicator, three indicator variables for the time periods (the 12 months prior to trauma, which served as the referent period, months 2–6, months 7–12, and months 13–36 after trauma), and all the interaction terms between these two sets of indicator variables. Second, among trauma patients only, we estimated with analogous GEE regression models the differences in mean number of days of sick leave according to either gender (male or female), age (20–29, 30–44, or 45–63 years), education (≤ 9, 10–12, or >12 years) or ISS (0–15, 16–24, 25–40, and 41–75) and time period (as previously defined). Age and educational level was set at the time of trauma and at the corresponding time for the matched controls. We also expressed the estimated mean differences in terms of Cohen’s *d*, a measure of effect size [16].

The association between potential risk factors and full-time sick leave month twelve following trauma was examined by logistic regression models. Associations are reported as odds ratios (OR) with corresponding 95% confidence intervals (CI). This analysis was carried out among the trauma patients after the exclusion of individuals deceased during the first year following trauma (*n* = 144). Age and gender, as well as patient-related (comorbidity, level of education, pre-trauma sick leave), trauma-related (ISS, GCS on arrival, AIS >2 for head- and spine-injury, systolic blood pressure on arrival), and treatment-related (hospital length of stay, discharge destination) risk factors were considered for this analysis. Missing data on education were noted for 114 patients. Gender, and all variables that were significantly associated with the outcome in univariate analyses, were included in a multivariable logistic model. The same multivariable model was also restricted to the sub-group of patients with an ISS > 15 (*n* = 880). A separate multivariable analysis was also done only including patients not on full-time sick leave for three consecutive months before trauma (*n* = 4522).

As a sensitivity analysis, we used probability weights in the multivariable logistic regression model to account for dropout from the study due to death [17]. The probability of death within 12 months after trauma was estimated with a logistic regression model including all the variables used in the multivariable analyses as well as year of trauma.

Data were analysed using SPSS Statistics 22.0 (SPSS Statistics IBM, Armonk, NY) and Stata/MP 14.2 (StataCorp, College Station, TX). All reported *P* values are two-sided and *P* values <0.05 were considered statistically significant.

## Results

The study population consisted of 4712 trauma patients and 25,013 controls. The median age of the trauma patients was 36 years and the majority, 3394 (72%) was male. Median ISS was 5 and approximately one-fifth was seriously injured (ISS > 15). General characteristics for trauma patients and controls are presented in Table 1. The difference in age distribution is due to the exclusion of trauma patients and controls receiving disability pension.

**Table 1:** Characteristics of the study population

	Trauma patients	Controls	*P* value^*^
Number of individuals	4712	25,013	
Age, years			0.025
20–29	1557 (33.0)	7862 (31.4)	
30–44	1667 (35.4)	8790 (35.1)	
45–63	1488 (31.6)	8361 (33.4)	
Gender			0.494
Female	1318 (28.0)	7119 (28.5)	
Male	3394 (72.0)	17,894 (71.5)	
History of comorbidity			
Psychiatric	523 (11.1)	1024 (4.1)	< 0.001
Substance abuse	609 (12.9)	617 (2.5)	< 0.001
Somatic	734 (15.6)	3116 (12.5)	< 0.001
Education			< 0.001
High	1200 (25.5)	9738 (38.9)	
Medium	2293 (48.7)	11,416 (45.6)	
Low	1105 (23.5)	3261 (13.0)	
Missing	114 (2.4)	598 (2.4)	
Sick leave month one before trauma, days/month			< 0.001
None	4440 (94.2)	24,320 (97.2)	
Part	187 (4.0)	495 (2.0)	
Full	85 (1.8)	198 (0.8)	
Sick leave month twelve after trauma, days/month			< 0.001
None	3747 (79.5)	24,239 (96.9)	
Part	326 (6.9)	418 (1.7)	
Full	495 (10.5)	334 (1.3)	
Dead	144 (3.1)	22 (0.1)	
Mechanism of injury			
Traffic-related	2752 (58.4)		
Fall	1032 (21.9)		
Assault	604 (12.8)		
Self-inflicted	134 (2.8)		
Other	188 (4.0)		
Missing	2 (0.0)		
Type of trauma			
Penetrating	317 (6.7)		
Blunt	4395 (93.3)		
ISS			
Median (IQR)	5 (1–13)		
≤ 15	3716 (78.9)		
> 15	996 (21.1)		
GCS on arrival			
14–15	3985 (84.6)		
9–13	313 (6.6)		
3–8	414 (8.8)		
AIS > 2			
Head	769 (16.3)		
Spine	217 (4.6)		
SAP on arrival, mm Hg			
≥ 90	4584 (97.3)		
< 90	128 (2.7)		
Length of stay hospital, days			
0–7	3756 (79.7)		
> 7	956 (20.3)		
Discharge destination			
Home	3745 (79.5)		
Other hospital/rehab	967 (20.5)		

Values in parentheses are percentages unless indicated otherwise

*ISS* Injury Severity Score, *GCS* Glasgow Coma Scale, *AIS* Abbreviated Injury Scale, *SAP* Systolic Arterial Pressure, *mm Hg* Millimeters of mercury

^*^χ^2^-test

Trauma patients had a higher prevalence of psychiatric comorbidity, substance abuse and somatic comorbidity. The trauma patients also demonstrated a lower level of education.

**Fig. 2:**
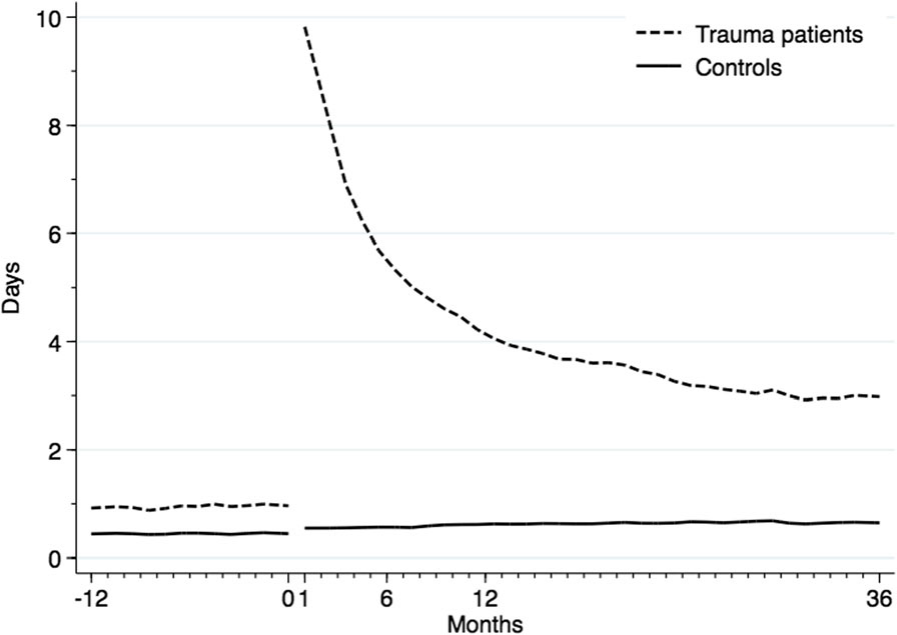
Characteristics of sick leave pre- and post-trauma. Sick leave over time measured as mean number of days per month in trauma patients and controls. Time of trauma depicted by time 0 on the x-axis

Compared to controls, the trauma cohort had a higher rate of sick leave the year preceding trauma, 0.9 days vs. 0.4 mean days/month (*p* < 0.001; Cohen’s d = 0.14). After trauma there was a marked increase in sick leave among trauma patients compared to controls. This difference declined but persisted (Fig. 2). During the period 7 to 12 months after trauma, the difference in mean days spent on sick leave between trauma and controls was 4.2 days/month (*p* < 0.001; Cohen’s d = 0.77). The difference in mean days spent on sick leave was still statistically significant in the period 13 to 36 months after trauma with a difference of 2.7 days/month (*p* < 0.001; Cohen’s d = 0.54).

**Fig. 3:**
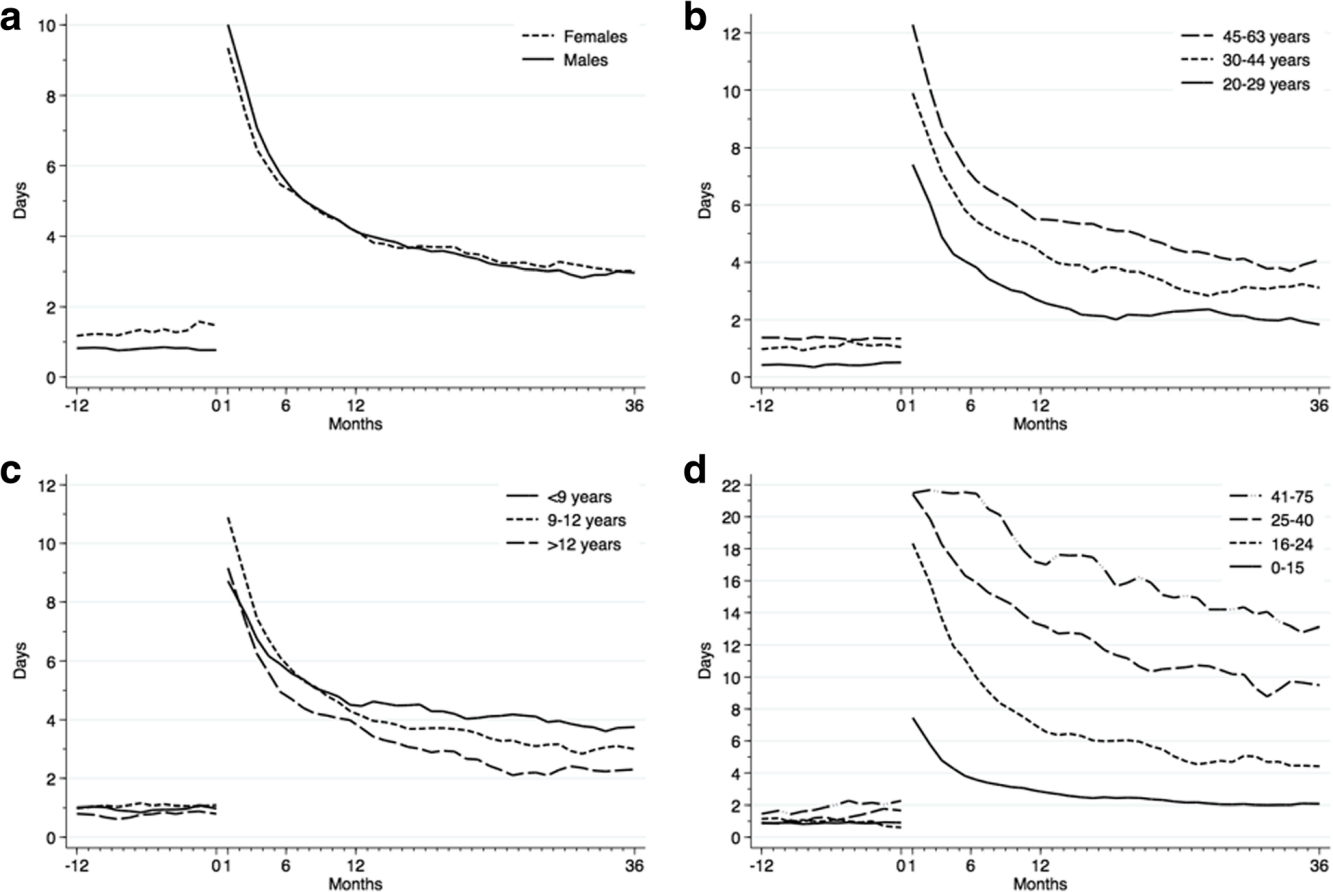
Mean number of days of sick leave in subgroups pre- and post-trauma. Sick leave over time measured as mean number of days per month pre- and post-trauma in subgroups of gender (**a**), age (**b**), years of education (**c**) and Injury Severity Score (**d**) among trauma patients. Time of trauma depicted by time 0 on the x-axis

The distribution of sick leave pre- and post-trauma in subgroups of gender, age, education and injury severity are displayed in Fig. 3. A higher rate of sick leave was noted in women and older patients prior to trauma. After trauma, sick leave was significantly lower in young patients, patients with less serious injury and higher education. No evidence of a difference between genders was noted after trauma.

Four hundred ninety-five trauma patients were on full sick leave month twelve after trauma, the primary end-point. In the multivariable regression analysis age, presence of psychiatric comorbidity, ISS 25–40, serious spinal injury (AIS spine >2), Glasgow Coma Scale (GCS) at admission <14, low and medium level of education, part- or full sick leave the month preceding trauma, discharge destination other than home, and hospital length of stay >7 days were all independently associated with full sick leave month twelve after trauma (Table 2). Pre-injury sick leave was strongly associated with the outcome variable. Results from the sensitivity analysis did not virtually change the results (data not shown).

**Table 2:** Odds ratios for full sick leave month twelve after trauma

	Univariate	*P* value*	Multivariable OR (95% CI)	*P* value^*^
Age, years		< 0.001		< 0.001
20–29	Ref		Ref	
30–44	1.82 (1.42–2.33)		1.76 (1.33–2.35)	
45–63	2.24 (1.75–2.88)		1.77 (1.32–2.38)	
Gender				
Female	Ref		Ref	
Male	1.19 (0.96–1.48)	0.109	1.08 (0.84–1.39)	0.564
History of comorbidity				
Psychiatric	2.21 (1.73–2.82)	< 0.001	1.46 (1.05–2.04)	0.024
Substance abuse	1.76 (1.38–2.25)	< 0.001	0.94 (0.68–1.29)	0.696
Somatic	1.46 (1.15–1.85)	0.002	1.05 (0.79–1.39)	0.753
Education		0.011		0.005
High	Ref		Ref	
Medium	1.30 (1.02–1.65)		1.36 (1.03–1.79)	
Low	1.51 (1.15–1.98)		1.70 (1.24–2.34)	
Sick leave month one before trauma, days/month		< 0.001		< 0.001
None	Ref		Ref	
Part	6.70 (4.88–9.19)		7.72 (5.30–11.23)	
Full	11.99 (7.59–18.94)		11.98 (7.04–20.39)	
ISS		< 0.001		0.025
≤ 15	Ref		Ref	
16–24	2.78 (2.15–3.59)		0.99 (0.70–1.40)	
25–40	7.45 (5.68–9.76)		1.67 (1.14–2.46)	
> 40	12.91 (7.73–21.57)		1.41 (0.73–2.72)	
GCS on arrival		< 0.001		< 0.001
14–15	Ref		Ref	
9–13	2.56 (1.88–3.50)		1.46 (1.00–2.13)	
3–8	6.71 (5.18–8.70)		2.40 (1.68–3.42)	
AIS > 2				
Head	3.80 (3.08–4.67)	< 0.001	1.17 (0.85–1.61)	0.327
Spine	4.39 (3.22–5.98)	< 0.001	2.36 (1.60–3.48)	< 0.001
SAP on arrival, mm Hg				
≥ 90	Ref		Ref	
< 90	3.32 (1.96–5.62)	< 0.001	0.81 (0.42–1.56)	0.526
Length of stay hospital, days				
0–7	Ref	< 0.001	Ref	< 0.001
> 7	6.26 (5.15–7.61)		2.68 (1.97–3.63)	
Discharge destination				
Home	Ref		Ref	
Other hospital/rehab	6.29 (5.17–7.65)	< 0.001	2.18 (1.64–2.89)	< 0.001

*OR* Odds Ratio, *CI* Confidence Interval, *ISS* Injury Severity Score, *GCS* Glasgow Coma Scale, *AIS* Abbreviated Injury Scale, *SAP* Systolic Arterial Pressure, *mm Hg* Millimeters of mercury

^*^χ^2^-test

**Fig. 4:**
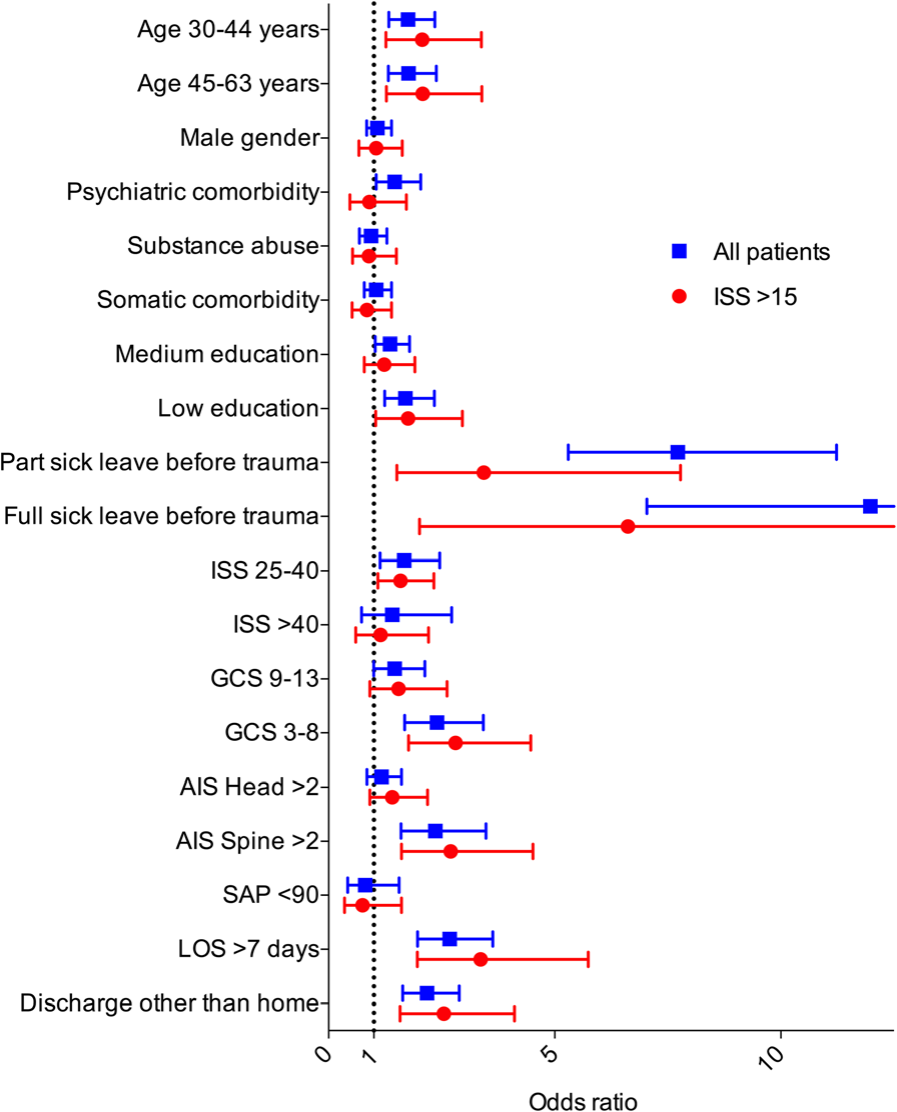
Odds ratios for full sick leave month twelve after trauma. Multivariable logistic regression analysis. Display of odds ratios with corresponding 95% confidence intervals. All patients (squares) and patients with ISS > 15 (dots). Injury Severity Score (ISS). Glasgow Coma Scale (GCS). Abbreviated Injury Scale (AIS). Systolic Arterial Blood Pressure (SAP, mmHg). Length of stay at hospital (LOS)

When restricting the analysis to the most seriously injured patients, i.e. ISS > 15 (of whom 230 out of 880 had the primary end-point), similar results were noted apart from that the presence of psychiatric comorbidity, GCS level 9–13 on admission and medium level of education were no longer significantly associated with full sick leave month twelve after trauma (Fig. 4).

In order to evaluate the effects of chronic pre-injury sick leave, individuals with full sick leave three consecutive months prior to injury were excluded from the analysis. This did not alter the main results.

## Discussion

In this retrospective matched observational cohort study we found a significant long-term morbidity, measured as sick leave, among trauma patients. Compared to controls the difference was maximal in the early phase after trauma and sustained throughout the follow up period of 36 months. Interestingly, the trauma patients took more sick leave the year before injury. In the logistic regression, factors associated with the traumatic injury per se as well as host factors increased the probability of not returning to work. Full sick leave month twelve post injury was strongly associated with pre-injury sick leave but also with age, psychiatric comorbidity, level of education, injury severity, spinal injury, low GCS at admission, length of stay at hospital and discharge to other destination than home.

Compared to other studies investigating the association between trauma and return to work, our cohort was less injured with a median ISS of 5 [2, 18–21]. However, one-fifth of the patients was seriously injured with an ISS > 15 and analysed separately, facilitating comparisons with previous studies.

Prior to injury the net sick leave was higher in trauma patients compared to controls. This can be explained by differences in baseline comorbidities, in particular the prevalence of psychiatric disease and substance abuse differed between the groups [22]. This finding is consistent with the notion that mental disorders are the most common diagnoses among those receiving compensation for sick leave and disability pension in Sweden [9]. Although the higher sick leave among trauma patients prior to trauma compared to controls to some extent was an expected finding, this association and its extent have previously not been investigated in trauma patients.

There are several studies reporting mortality after trauma [23–26], but there is a gap of knowledge regarding outcome in terms of morbidity. The current study investigates sustained morbidity as reflected by prolonged sick leave after trauma. The long-term impact of trauma on sick leave is highlighted by the fact that sick leave rates did not return to baseline during the entire study period. In a previous Australian study, a large proportion of those with serious injury had at least moderate disability at 24 months follow up [2] in line with our findings. Of those with reported sick leave month 36 after trauma in the present study, a majority (61%) was on full sick leave. In the absence of information regarding the cause of sick leave at an individual level, we cannot fully analyse the reasons behind the residual increase. It could be caused by individuals never returning to work because of traumatic sequelae but obviously also by factors including other diagnoses not related to the trauma.

Male gender was not a risk factor for full sick leave month twelve after trauma. This is somewhat surprising considering that previous studies have indicated that male gender is a risk factor for worse outcome after trauma, although the impact of gender has been debated [27–32]. Females had a significantly higher rate of sick leave before trauma compared to males, a difference that was omitted after trauma suggesting that males might be more affected by trauma in terms of sick leave. This finding is somewhat in contrast to the study by Gabbe et al. reporting female gender as a risk factor for delayed return to work after trauma [2]. As expected, older individuals had more days of sick leave both before trauma and throughout the study period. A low socioeconomic position is associated with an increased risk of becoming a trauma victim ([22, 33, 34]. The influence of socio-economy was also apparent in the current study where a high level of education was associated with less sick leave, which is in line with previous studies reporting high education as protective for functional outcome after trauma [35]. This finding has been suggested to relate to compliance with treatment and a high degree of white collar occupations [36].

Not surprisingly, serious spinal injury and serious injury to the head were associated with a higher probability of being on full-time sick leave month twelve after trauma, although the results for serious head injury did not reach statistical significance. The latter finding could be partly explained by the fact that serious head injury was associated with a higher probability of death within one year from trauma as compared with non-serious head injury. Thus, patients with serious head injury were less likely to be included in the analyses for the endpoint sick leave month twelve after trauma. However, the fact that the results from the sensitivity analysis were similar to those from the main analyses indicates that other factors might explain the lack of statistical significance. The fact that low GCS on arrival was rather strongly associated with the endpoint could be due to that consciousness not only reflects head injury but also other conditions such as intoxication and shock. As expected injury severity was associated with full sick leave month twelve after trauma. The lack of a significant association for patients exceeding ISS 40 is likely due a high probability of death yielding a limited number of patients (*n* = 62) twelve months after trauma. Length of stay at hospital and discharge to a destination other than home were both associated with a high rate of sick leave. Most likely these parameters summarize the total need for recovery and rehabilitation of trauma patients.

A major finding in the current study was the strong association between pre- and post-injury sick leave. Disability as well as sick leave is well known to be associated with circumstances not limited to disease or injury severity per se. Several factors such as working conditions, ethnicity, residency, gender, education and income may have a significant influence on sick leave according to recent official Swedish reports [37]. In summary, this indicates that pre-injury sick leave might serve as a proxy for accumulated risk factors that together make these individuals more prone to be on sick leave, both before and after trauma.

The trauma cohort in the current study had a fairly low median ISS, suggesting that other factors than injury severity would have a strong impact on the outcome. However, when restricting the analyses to patients with more serious injury (ISS > 15) only a small difference in risk factors was noted. In fact, the only major change was that psychiatric disease no longer was associated with sick leave. This finding indicates that non-injury related risk factors are highly important also in the most seriously injured patients.

It is important to emphasize that the number of days of sick leave is affected by the current social security system influencing comparisons with other settings and populations. Regardless of system, sick leave is closely connected to functional status and morbidity. Knowledge of risk factors may allow for future models predicting sustained morbidity measured as sick leave, a potentially important clinical tool for preventive measures such as targeted rehabilitation.

Not all episodes of sick leave less than 14 days are recorded in the Swedish Social Insurance Agency database. Because of this the first month after trauma was excluded from the analysis. The peak of reported sick leave in this study occurs the second month after trauma, even though it is fair to conclude that the first month after trauma contains the same or even higher numbers of sick leave.

There are several limitations to the present study. External validity might be reduced by the single-centre approach. Other limitations are associated with the retrospective, register-based study design, whereas the use of validated national health registries is considered a strength [12]. There was a median age of 36, a dominance of males and approximately one third of patients exhibiting some pre-existing medical condition. The demography is therefore in line with several other trauma studies [38–40]. Data on causes of sick leave is not available limiting the interpretation of the findings. Differences in social insurance systems between countries may also limit the reproducibility of the results. A very low rate of missing data and minimal loss to follow-up also strengthen the study. Emigration during the study period could influence loss to follow up and the risk of obtaining sick leave, however we have no reason to believe that there is a difference between exposed (trauma patients) and unexposed (uninjured patients). The annual national emigration rate was less than 0.5% during the study period and is considered to be of minor importance.

## Conclusions

In this large retrospective matched observational cohort study we found a sustained long-term morbidity among trauma patients throughout the follow up period of three years. Non-trauma related factors, in particular pre-injury sick leave, had a significant influence on the study endpoint, full sick leave month twelve after injury. An increase in sick leave among trauma patients compared to controls was noted also before the time of trauma. Although sick leave is influenced by pre-existent and demographic factors, it may serve as one of the quantifying parameters for post trauma morbidity.

## Abbreviations

AIS: Abbreviated injury scale
CI: Confidence intervals
GCS: Glasgow coma scale
ICD-10: International classification of diseases
ISS: Injury severity score
LOS: Length of stay at hospital
NBHW: National board of health and welfare
OR: Odds ratios
SAP: Systolic arterial blood pressure
STROBE: Strengthening the reporting of observational studies in epidemiology
